# ConCysFind: a pipeline tool to predict conserved amino acids of protein sequences across the plant kingdom

**DOI:** 10.1186/s12859-020-03749-2

**Published:** 2020-10-31

**Authors:** Marten Moore, Corinna Wesemann, Nikolaj Gossmann, Arne Sahm, Jan Krüger, Alexander Sczyrba, Karl-Josef Dietz

**Affiliations:** 1 Biochemistry and Physiology of Plants, Bielefeld University, 33501 Bielefeld, Germany; 2 Computational Biology Group, Leibniz Institute on Aging – Fritz Lipmann Institute, Jena, Germany; 3 Computational Metagenomics, Bielefeld University, 33501 Bielefeld, Germany

**Keywords:** Alga, Conservation, Cysteine, Evolution, Phylogeny, Plants, Protein, Redox regulation, Translation

## Abstract

**Background:**

Post-translational modifications (PTM) of amino acid (AA) side chains in peptides control protein structure and functionality. PTMs depend on the specific AA characteristics. The reactivity of cysteine thiol-based PTMs are unique among all proteinaceous AA. This pipeline aims to ease the identification of conserved AA of polypeptides or protein families based on the phylogenetic occurrence in the plant kingdom. The tool is customizable to include any species. The degree of AA conservation is taken as indicator for structural and functional significance, especially for PTM-based regulation. Further, this pipeline tool gives insight into the evolution of these potentially regulatory important peptides.

**Results:**

The web-based or stand-alone pipeline tool Conserved Cysteine Finder (ConCysFind) was developed to identify conserved AA such as cysteine, tryptophan, serine, threonine, tyrosin and methionine. ConCysFind evaluates multiple alignments considering the proteome of 21 plant species. This exemplar study focused on Cys as evolutionarily conserved target for multiple redox PTM. Phylogenetic trees and tables with the compressed results of the scoring algorithm are generated for each Cys in the query polypeptide. Analysis of 33 translation elongation and release factors alongside of known redox proteins from *Arabidopsis thaliana* for conserved Cys residues confirmed the suitability of the tool for identifying conserved and functional PTM sites. Exemplarily, the redox sensitivity of cysteines in the eukaryotic release factor 1-1 (eRF1-1) was experimentally validated.

**Conclusion:**

ConCysFind is a valuable tool for prediction of new potential protein PTM targets in a broad spectrum of species, based on conserved AA throughout the plant kingdom. The identified targets were successfully verified through protein biochemical assays. The pipeline is universally applicable to other phylogenetic branches by customization of the database.

## Background

Post-translational modifications (PTM) represent highly dynamic and often reversible mechanisms to alter protein properties by adding or modifying a chemical group at one or multiple amino acids (AA). PTMs diversify the structure and function of a single polypeptide enormously. They allow adopting multiple regulatory states ranging from switching on/off or tuning their respective activity, altering the stability or even acquiring new functions, e.g., by moonlighting in cellular signal transduction [1]. Amino acids available for PTMs often are part of functional domains with conserved sequence environment. These domains can be aligned between the cognate representatives from different species and unravel evolutionary events if placed in phylogenetic context, e.g. by use of a phylogenetic tree. A particular AA appropriate for PTM emerging during evolution and increasing fitness likely is maintained in descendants.

Protein phosphorylation was one of the first PTMs reported in literature in context of glycogen degradation [2]. It describes the addition of a phosphate moiety to the hydroxyl group of serine, threonine or tyrosine residues, but may also occur at other residues including histidine, aspartate and cysteine [3]. Regulated by antagonistically acting protein kinases and phosphatases, it controls key cellular processes, especially in intracellular and cell-to-cell communication and coordination of cellular metabolism. Redox-PTMs describe the process of reduction and oxidation of target proteins and involve thiol groups of cysteinyl residues, but also methionine sulfoxide formation. Cysteinyl thiols can oxidise to disulfides, sulfenic, sulfinic or sulfonic acid derivatives, but also to *S*-glutathionylated, persulfidated, *S*-nitrosylated and other forms [4]. These modifications can affect tertiary and quaternary structure, binding abilities and activities of the proteins [5–9]. Thus, thiol redox regulation is a prominent PTM involved in most cellular processes in plants, like photosynthesis, lipid synthesis, gene expression, cell cycle control and protein biosynthesis [10, 11].

PTMs depend on AA side chains accessible for catalytic interaction partners or substrates. In case of redox regulation, such partners are thioredoxins (Trx), peroxiredoxins (Prx) and H_2_O_2_ [12]. If beneficial for the organism, such a regulatory mechanism serves as blueprint and is recognizable by conserved sequence environments during subsequent evolution. By constructing multiple alignments between protein homologues from different evolutionarily distinct species, conserved sequence domains and especially PTM-sensitive AA hint towards functional and structural similarities. Here, we present a stand-alone and web server tool that identifies conserved AA needed for redox regulation or phosphorylation by comparing the query sequence with the most related sequences featuring the target AA from 21 species selected from the plant kingdom: Conserved Cysteine Finder (ConCysFind) (Additional file 1: Figure 1). Utilizing this approach, ConCysFind with its flow diagram as depicted in Additional file 1: Figure 2 represents the first universally extendable pipeline for PTM site identification based on phylogeny, allowing the user an easy and reliable in silico prediction independent on often limited mass spectrometry data sets and treatment conditions. As exemplar study, we decided to investigate translation elongation and termination factors for conserved cysteines, since research on protein synthesis is mostly focused on translation initiation and phosphorylation as PTM with neglection of redox-PTM [13]. Translation is a concerted and complex cellular process which affects growth, differentiation and stress response. All three major steps of eukaryotic translation, namely initiation, elongation and termination, are realised and controlled by so-called eukaryotic translation factors, which underlie several levels of regulation.

Eukaryotic initiation factors (eIF) and eukaryotic elongation factors (eEF) are consistently described as targets of Cys-based PTM in several independent studies [14–17]. However, redox regulation has not gained the same acceptance as regulatory mechanism as phosphorylation so far. Here we show that besides initiation and elongation, termination features the potential for redox regulation via Cys-PTM. The automated and systematic exploration of conserved Cys or other AA enables a fast screening for possibly redox- or phosphorylation-based regulation of proteins and directs research for validation by wet lab analyses as shown here for the eukaryotic release factor 1–1 (eRF1-1) from *A. thaliana*.

## Implementation

ConCysFind is a Java-based pipeline tool that utilizes BioJava [18] as a web-based tool accessible at BiBiServ2 (https://bibiserv.cebitec.uni-bielefeld.de/concysfind), or as local tool following its download and execution on Windows, macOS or Linux systems. The input sequences are pasted in the online tool as Uniprot ID as the first column and an optional protein description in the second column. Another possibility is uploading the query sequences as tab-separated value format (.tsv) file. According to our aim to study thiol regulation in plants based on the plant-related *Tree of Life Web Project* (https://tolweb.org/tree/) [19], 21 species from algae to higher plants, including *Arabidopsis thaliana, Beta vulgaris, Zea mays* and *Oryza sativa* (see Additional file 1: Figure 1) were selected. The available protein sequences were assembled as the tool’s default protein database with proteome sequences from UniProt (https://www.uniprot.org/) (The UniProt Consortium 2017) (as available in December 2018) [20]. We selected species that represent high evolutionary diversity and are evenly spread among the different plant taxa, under consideration of one proxy species per species.

Custom databases from any organism can be compiled and added in.fasta format via BLAST+ [21] (see Additional Methods and Handbook). The number of selected species determines the run time and storage space requirements of the multiple alignments and should be taken into account, especially if ConCysFind is executed on local systems. In line with run-time limits of local machines and operating systems, but with the option to use large protein families as input, we selected the BioJava platform with its reliably cross platform running algorithm.

To generate a multiple alignment, the pipeline tool uses blastp to select a maximum of 9 closest homologues per query in each species defined in the database, based on their respective blast-score and e-value. This appeared important in order to exclude that the Cys only is conserved in some homologues but was lost during evolution in other homologues, e.g., following gene duplication and neofunctionalization. Therefore in a unique manner, the blastp-alignment and AA identification routine is run in up to 9 iterations for the same species, starting with the most similar sequence based on the whole protein blast results and incrementing if the AA is not detected (see Additional file 1: Figure 2 BLAST).

The BLAST result-selected candidates with high sequence similarity allowed the usage of runtime-efficient alignment algorithms to generate a global multiple alignment utilizing a heuristic greedy algorithm with a BLOSUM62 cost matrix [22]. Subsequently, AA score and *p *value are computed for each Cys. The *p *value determination incorporates on the one hand the frequency of occurrence of Cys at one particular position and on the other hand the degree of conservation of the total protein. By consideration of the global conservation of the AA sequence, and therefore conserved features of the polypeptide in total in conjunction with the conserved Cys position, a strong indication of functional significance can be assumed. Since indiscriminate introduction of Cys underlies strong counter selection, even Cys residues without direct phylogenetic relation are captured in the score, expecting the presence of Cys rarely occurs randomly without functional advantage. Based on the multiple alignments, ConCysFind constructs phylogenetic trees for each Cys following the Neighbour Joining Algorithm [23] using the forester library, which exports the phylogenetic tree in PNG-format.

The export of the generated phylogenetic trees is not possible on the Solaris-Server hosting the BiBiServ2 platform, instead a tree for each Cys is given in Newick-Strings annotation in a.txt file. The trees are featured in the download version of ConCysFind. The output is handled by the org.apache.commons.cli apache package, converting the.txt files to.xls files for easier handling. The complete output consists of a log-file, an excel-table with scores and *p *values, the.txt-file with the multiple alignments and a folder containing the phylogenetic trees for each Cys of the input sequence. The additional handbook provides all parameters. Users can customise these parameters according to their preferences (see Additional file 1: Methods and Handbook).

## Results and discussion

For testing ConCysFind we chose a process that is under-investigated in terms of redox regulation, namely protein synthesis [13]. In addition to testing unknown proteins, we used an established test set of known redox proteins. We compiled the.tsv file consisting of UniProt entries from all known translation factors in *Arabidopsis thaliana* (Additional file 1: Table 1) and added the redox network components peroxiredoxin (Prx) IIB, 2-Cys Prx B, Thioredoxin (Trx)-f, Trx-h as well as SAL1 phosphatase [24, 25]. Analysis of 33 translation factors and redox regulators for conserved Cys with the standard parameter settings of ConCysFind (see Additional file 1: Tables 2 and 3) revealed a total of 169 Cys in 33 protein sequences, carrying a total of 114 conserved Cys (*p* ≤ 0.01) (see Table 1). Literature on all investigated proteins was queried for relevant Cys-based PTMs employing different quantitative redox-proteomic approaches (see Table 1, Additional file 1: Table 1).

**Table 1:** Excerption table of results

Name	UniProt ID	Homologs	Putatively conserved	Cys position	Cys score (*p *value)	Cys-PTM
eEF1α	Q8GTY0	19	6 of 6	Cys1: 87	0.53 (9.09e^−4^)	S-NO, S-H_2_, oxidation
				Cys2: 111	0.53 (9.09e^−4^)	
				Cys3: 150	0.58 (1.07e^−4^)	
				Cys4: 151	0.53 (9.09e^−4^)	
				Cys5: 152	0.53 (9.09e^−4^)	
				Cys6: 351	0.68 (1.00e^−6^)	
eEF1β	Q9SCX3	13	1 of 1	Cys1: 216	0.77 (2.45e^−3^)	S-NO
eEF1γ	Q9FVT2	14	3 of 4	Cys1: 165	0.57 (7.71e^−3^)	S-NO, S–S
				Cys2: 303	0.71 (1.03e^−4^)	Oxidation
				Cys4: 411	0.50 (3.87e^−2^)	
eEF1δ	P48006	13	1 of 2	Cys2: 223	0.77 (1.44e^−3^)	
eEF1A	Q8L835	19	3 of 5	Cys1: 305	0.95 (1.32e^−7^)	S-NO
				Cys3: 454	0.79 (2.84e^−4^)	
				Cys5: 495	0.89 (3.00e^−6^)	
eEF1B	A8MRC4	13	1 of 4	Cys2: 223	0.77 (5.45e^−3^)	
eEF2	Q9ASR1	19	14 of 16	Cys1: 131	0.95 (7.84e^−16^)	S-NO, S-SG
				Cys2: 136	0.95 (7.84e^−16^)	S–S, oxidation
				Cys3: 163	0.84 (4.16e^−12^)	
				Cys4: 267	0.84 (4.16e^−12^)	
				Cys5: 275	0.53 (1.11e^−4^)	
				Cys6: 286	0.63 (1.00e^−6^)	
				Cys7: 370	1.00 (3.27e^−18^)	
				Cys8: 448	0.84 (4.16e^−12^)	
				Cys9: 495	0.74 (3.90e^−9^)	
				Cys10: 520	0.89 (7.55e^−14^)	
				Cys11: 541	0.95 (7.84e^−16^)	
				Cys13: 651	0.74 (3.90e^−9^)	
				Cys15: 690	0.58 (1.20e^−5^)	
				Cys16: 797	0.89 (7.55e^−14^)	
eRF1-1	Q39097	18	3 of 3	Cys1: 126	1.00 (1.13e^−13^)	Oxidation
				Cys2: 388	0.89 (7.50e^−10^)	
				Cys3: 404	0.78 (3.75e^−7^)	
2CP	Q96291	18	2 of 2	Cys1: 119	1.00 (3.92e^−12^)	S-NO, S-SG
				Cys2: 241	0.89 (1.97e^−8^)	S–S, oxidation
SAL1	Q42546	18	3 of 4	Cys1:21	0.67 (0.00173)	S–S, oxidation
				Cys3:167	1.00 (2.62e^−10^)	
				Cys4:190	0.611 (0.008)	

Excerption of the results table (Additional file 2: Table 1) generated via ConCysFind with the input query (Additional file 1: Table 2) and run with default parameters. Shown are UniProt ID, the number of homologs identified among the 21 selected species, position and scoring of conserved cysteines as well as identified Cys-PTM for every protein found in literature

**Fig. 1:**
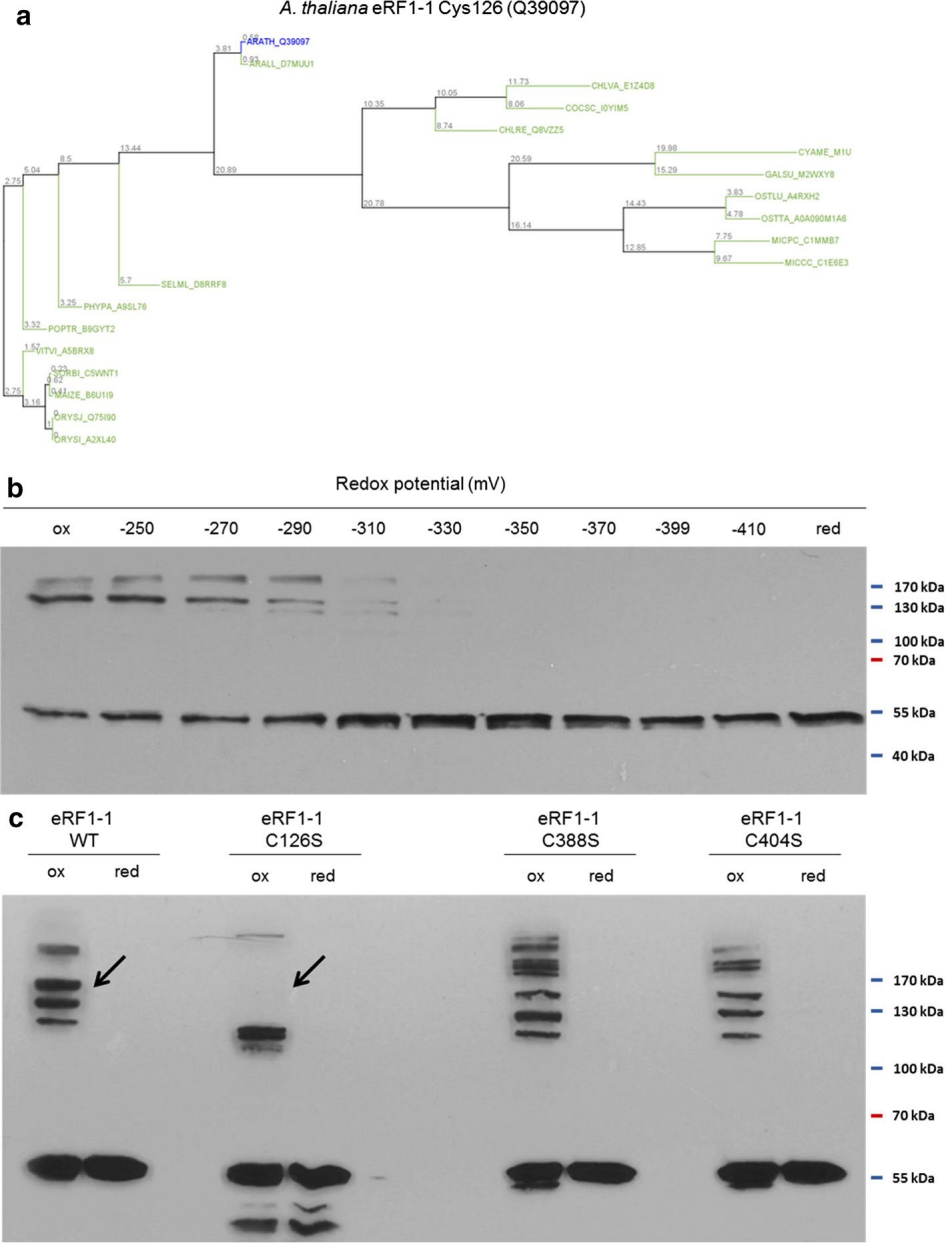
eRF1-1 shows Cys126-centered redox sensitivity in vitro. **a** Phylogenetic tree of Cys126 of *A. thaliana* eRF1-1 as example for ConCysFind output trees. The phylogenetic tree represents the grade of similarity between the most similar protein sequences, found in each of the 21 proteomes compared to the input sequence of eRF1-1. Thus it has a phylogenetic aspect and indicates functional significance. eRF1-1 Cys126 represents a newly identified fully conserved cysteine in the plant kingdom, indicating a potential redox-sensitive functionality in vivo for this particular residue. **b** Western Blot of eRF1-1 in redox gradient. eRF1-1 was subjected to distinct ratios of DTT_ox_ and DTT_red_, spanning from fully oxidising (≥ 250 mV) to fully reducing (≤ 410 mV) conditions. Besides the eRF1-1-His_6_ monomer (ca. 50 kDa), eRF1-1 oligomers were visualised with anti His_6_-antibody. **c** eRF1-1 wildtype protein and Cys-to-Ser variants C126S, C388S and C404S under fully oxidising (ox) and reducing (red) conditions after Western blotting and detection with anti His_6_-antibody. Significant differences in oligomerisation pattern under oxidising conditions are indicated with black arrows

Of the previously characterised cysteines, Cys241 and Cys119 of 2-Cysteine Peroxiredoxin (2CP) and Cys21, Cys167 and Cys190 of SAL1 phosphatase (SAL1) were correctly predicted as conserved (Table 1, Additional file 1: Figure 3). In fact, ConCysFind detected all previously described conserved and functional Cys-residues in Prxs and Trxs as well as the translation factor subset. Importantly, the tool identified other conserved, so far uncharacterized redox-regulated Cys, emphasising the predictive power of ConCysFind. eRF1-1 was among the previously uncharacterised proteins. Especially Cys126 of eRF1-1 was conserved with a very high score (Table 1, Fig. 1a). The conservation of this specific Cys across the 21 proteomes lead to the assumption that the evolutionary conservation of this particular Cys aligns with the conservation of structure and function of the protein in question.

To test the assumption that the newly identified conserved Cys also serve as PTM sites, we selected eRF1-1 for in vitro validation. To this end, wildtype *A. thaliana* eRF1-1 (UniProt ID: Q39097) and three Cys-to-Ser variants were generated, heterologously expressed in *E. coli*, purified and subjected to different redox environments adjusted by redox buffers (see Fig. 1b, c). The physiological thiol redox state of the cytosol ranges between − 270 mV (oxidising), − 310 (resting state) and − 330 mV (over-reducing condition) [26]. eRF1, together with eRF3, terminates protein synthesis by stop codon recognition and hydrolysis of the ester bond linking the polypeptide chain to the final peptidyl-tRNA [27, 28].

*Arabidopsis thaliana* eRF1-1 carries three Cys, all of which are conserved, but without previous link to redox susceptibility or regulation. The accessibility, redox-sensitivity and possible regulatory function of Cys can be scrutinized by redox titration in vitro. Recombinant protein was exposed to redox buffers adjusting physiologically relevant redox potentials in the range of − 250 mV as oxidizing and − 410 mV as reducing condition. Intra- or intermolecular dithiol-disulfide transitions are verifiable by band shifts in SDS–polyacrylamide gel electrophoretic separations.

The redox titration revealed a prominent and relevant redox shift of eRF1-1 between − 290 and − 330 mV, indicating thiol redox changes in the physiological redox potential range of the cytosol [26]. We substituted Ser for each Cys of eRF1-1, generating the variants C126S, C388S and C404S. The variant proteins revealed slightly altered mobility in all cases relative to the WT form as visualized by additional bands for Cys388 and Cys404 of dimers or oligomers under oxidizing conditions if separated by non-reducing SDS-PAGE. The most pronounced change occurred for the variant C126S, which adopted the monomeric and dimeric form. But all bands with higher molecular mass could not be detected. Oligomerisation might provide a short- or medium-term holding mechanism for translation termination, by sterically blocking eRF1–eRF3 interaction and therefore GTP-stimulated hydrolysis of the polypeptide chain.

ConCysFind classified all three Cys of eRF1-1 as conserved AA by using default settings. However, Cys388 (Cys-Score: 0.88) and Cys404 (Cys-Score: 0.77) are present in the green lineage with few exceptions, pointing to regulatory mechanisms evolved in photosynthesis (see Additional file 1: Figure 4). In a converse manner, Cys126 shows global conservation (Cys-Score: 1.0) even beyond the investigated plant species, as it is present at the same relative position in mammals [29]. Therefore, Cys126 presumably represents a conserved key feature involved in a general regulatory mechanism of eRF1-1.

## Conclusions

ConCysFind grants easy access to evolutionarily conserved AA in protein families. This simplifies the selection criteria for experimental biologist and helps elucidating possible functional residues, domains and structures. Commonly encountered PTMs concern the AA Ser, Thr and Tyr for phosphorylation, and Cys and Met for sulfur modifications. The phylogenetic tree visualisation of each analysed AA augments the conclusions beyond *p *value calculation to the level of understanding evolution. The tool addresses the question when during evolution a particularly regulatory mechanism emerged. Discovery of new regulatory PTM-elements advances our understanding of the functionality of a given protein of interest. The chosen example eRF1-1 has not been investigated as redox target before. As a matter of fact, since the work cited in references (1987 and 1992), much progress has been made.

The progressive greedy algorithm reliably worked with our test proteins and translation factors. In future work, it should be tested whether parsimony and maximum likelihood methods allows to improve the results when it comes to sequences with lower similarity [30, 31]. The pipeline tool provides a versatile and easy to use approach to analyse proteins in silico, potentially revealing novel regulatory elements in single proteins or protein families of interest. The web browser version of ConCysFind will be further improved, based on user’s feedback and the database updated and maintained at https://bibiserv.cebitec.uni-bielefeld.de/concysfind.

## Availability and implementation

- Project name: ConCysFind.
- Project home page: https://bibiserv.cebitec.uni-bielefeld.de/concysfind.
- Operating system(s): either BibiServ2 or Platform independent.
- Programming language: Java.
- Other requirements: Java RE8 or newer; for local use: Linux, Mac and Windows OS.
- License: Not applicable.
- Any restrictions to use by non-academics: No restrictions.

## Supplementary information

**Additional file 1: Figure 1.** Phylogenetic tree of the plant species in database. The genomes of the given species are included in the default setting of ConCysFind. The phylogenetic tree of the selected 21 species was constructed following the Tree of Life Web Project [19] (https://tolweb.org/tree/). The species are depicted as leaves with taxon description at the inner nodes. Here, the edges do not relate to evolutionary distances. **Figure 2. **Flow diagram of ConCysFind. Depicted are the working steps of the pipeline tool ConCysFind, sub-sectioned by the category of process. Input can be a single protein sequence in single letter code or multiple protein queries as UniProt ID and description in.tsv format. Optionally to the default database, a custom database of 21 species can be used. Each protein sequence *1*…*i* is BLASTed for each species against the database resulting in a maximum of *x* BLAST hits per sequence *i*. A nine-times iteration is executed, eliminating close blast hits that do not carry the query AA with a BLAST score cut-off of 0.8 to obtain the nine best BLAST hits per species. A multiple alignment of each sequence *i* and all species is generated for each stored BLAST sequence. Following the multiple alignments, a phylogenetic tree for the whole protein sequence of each sequence *i* is constructed and subsequently the *p *value and score for each analysed AA is calculated. Lastly, for each AA a phylogenetic tree representing the individual conservation-score and *p *value is drawn. The outputs of the alignments and results are stored as.txt and the phylogenetic trees as.png in a subfolder. **Figure 3.** Output example of phylogenetic trees with conserved cysteines of known redox targets. (A) Phylogenetic tree of fully conserved Cys167 of the SAL1 phosphatase, a previously characterised target of functional oxidation and disulphide formation [24]. (B) Partial conservation of Cys241 of chloroplast redox sensor 2-cysteine peroxiredoxin in 21 plant species. **Figure 4.** Phylogenetic trees of partially conserved Cys388 and Cys404 of eRF1-1. (A) Cys388 is fully conserved within the green lineage, but not in red algae *C. merolae* and *G. sulphuraria*, resulting in 88% overall conservation. (B) With 77% overall conservation, Cys404 is the least conserved Cys of eRF1-1, as it is missing in red algae and two *Micromonas* representatives. **Table 2.** Input query. Consisting of 33 UniProt ID and their descriptions of translation elongation and termination factors, as well as 6 proteins previously described as being PTM-regulated. **Table 3.** ConCysFind parameters.

**Additional file 2: Table 1.** Result scores for all translation factors and selected known redox targets of *A. thaliana*. See separate xlsx-file.

## Data Availability

All data generated or analysed during this study are included in this published article and its additional information files.

## Abbreviations

AA: Amino acid
ConCysFind: Conserved Cysteine Finder
*E. coli*: *Escherichia coli*
eIF: Eukaryotic initiation factor
eEF: Eukaryotic elongation factor
eRF: Eukaryotic release factor
Prx: Peroxiredoxin
PTM: Post-translational modification
SAL1: SAL1 phosphatase
Trx: Thioredoxin

## References

[1] Bensimon A, Heck AJR, Aebersold R (2012). Mass spectrometry-based proteomics and network biology. Annu Rev Biochem.

[2] Nimmo HG, Proud CG, Cohen P (1976). The phosphorylation of rabbit skeletal muscle glycogen synthase by glycogen synthase kinase-2 and adenosine-3':5'-monophosphate-dependent protein kinase. Eur J Biochem.

[3] Hunter T (2012). Why nature chose phosphate to modify proteins. Philos Trans R Soc Lond B Biol Sci.

[4] Biswas S, Chida AS, Rahman I (2006). Redox modifications of protein-thiols: emerging roles in cell signaling. Biochem Pharmacol.

[5] Pejaver V, Hsu W-L, Xin F, Dunker AK, Uversky VN, Radivojac P (2014). The structural and functional signatures of proteins that undergo multiple events of post-translational modification. Protein Sci.

[6] Henze A, Homann T, Serteser M, Can O, Sezgin O, Coskun A (2015). Post-translational modifications of transthyretin affect the triiodonine-binding potential. J Cell Mol Med.

[7] Bah A, Forman-Kay JD (2016). Modulation of intrinsically disordered protein function by post-translational modifications. J Biol Chem.

[8] Liebthal M, Maynard D, Dietz K-J (2018). Peroxiredoxins and redox signaling in plants. Antioxid Redox Signal.

[9] Tolsma TO, Hansen JC (2019). Post-translational modifications and chromatin dynamics. Essays Biochem.

[10] Buchanan BB, Balmer Y (2005). Redox regulation: a broadening horizon. Annu Rev Plant Biol.

[11] Dietz K-J (2008). Redox signal integration: from stimulus to networks and genes. Physiol Plant.

[12] Vaseghi M-J, Chibani K, Telman W, Liebthal MF, Gerken M, Schnitzer H (2018). The chloroplast 2-cysteine peroxiredoxin functions as thioredoxin oxidase in redox regulation of chloroplast metabolism. Elife.

[13] Moore M, Gossmann N, Dietz K-J (2016). Redox REGULATION OF CYTOSOLIC TRANSLATION IN PLANTs. Trends Plant Sci.

[14] Lindermayr C, Saalbach G, Durner J (2005). Proteomic identification of S-nitrosylated proteins in Arabidopsis. Plant Physiol.

[15] Wang H, Wang S, Lu Y, Alvarez S, Hicks LM, Ge X, Xia Y (2012). Proteomic analysis of early-responsive redox-sensitive proteins in Arabidopsis. J Proteome Res.

[16] Liu P, Zhang H, Wang H, Xia Y (2014). Identification of redox-sensitive cysteines in the Arabidopsis proteome using OxiTRAQ, a quantitative redox proteomics method. Proteomics.

[17] Aroca Á, Serna A, Gotor C, Romero LC (2015). S-sulfhydration: a cysteine posttranslational modification in plant systems. Plant Physiol.

[18] Prlić A, Yates A, Bliven SE, Rose PW, Jacobsen J, Troshin PV (2012). BioJava: an open-source framework for bioinformatics in 2012. Bioinformatics.

[19] Maddison DR, Schulz K-S, Maddison WP (2007). The tree of life web project. ZOOTAXA.

[20] The UniProt Consortium (2017). UniProt: the universal protein knowledgebase. Nucleic Acids Res.

[21] Camacho C, Coulouris G, Avagyan V, Ma N, Papadopoulos J, Bealer K, Madden TL (2009). BLAST+: architecture and applications. BMC Bioinform.

[22] Henikoff S, Henikoff JG (1992). Amino acid substitution matrices from protein blocks. Proc Natl Acad Sci U S A.

[23] Saitou N, Nei M (1987). The neighbor-joining method: a new method for reconstructing phylogenetic trees. Mol Biol Evol.

[24] Chan KX, Mabbitt PD, Phua SY, Mueller JW, Nisar N, Gigolashvili T (2016). Sensing and signaling of oxidative stress in chloroplasts by inactivation of the SAL1 phosphoadenosine phosphatase. Proc Natl Acad Sci U S A.

[25] Gerken M, Kakorin S, Chibani K, Dietz K-J (2020). Computational simulation of the reactive oxygen species and redox network in the regulation of chloroplast metabolism. PLoS Comput Biol.

[26] Schwarzländer M, Fricker MD, Müller C, Marty L, Brach T, Novak J (2008). Confocal imaging of glutathione redox potential in living plant cells. J Microsc.

[27] Alkalaeva EZ, Pisarev AV, Frolova LY, Kisselev LL, Pestova TV (2006). In vitro reconstitution of eukaryotic translation reveals cooperativity between release factors eRF1 and eRF3. Cell.

[28] Jackson RJ, Hellen CUT, Pestova TV (2012). Termination and post-termination events in eukaryotic translation. Adv Protein Chem Struct Biol.

[29] Cheng Z, Saito K, Pisarev AV, Wada M, Pisareva VP, Pestova TV (2009). Structural insights into eRF3 and stop codon recognition by eRF1. Genes Dev.

[30] Hall BG (2005). Comparison of the accuracies of several phylogenetic methods using protein and DNA sequences. Mol Biol Evol.

[31] Katoh K, Kuma KI, Toh H, Miyata T (2005). MAFFT version 5: improvement in accuracy of multiple sequence alignment. Nucleic Acids Res.

