# Supplementary material for: ConCysFind: a pipeline tool to predict conserved amino acids of protein sequences across the plant kingdom
Source: BMC Bioinformatics. 2020 Oct 31;21:490. doi: 10.1186/s12859-020-03749-2 (PMC7603750; doi:10.1186/s12859-020-03749-2)
Supplement: Supplementary file 1 — Additional file 1: Figure 1. Phylogenetic tree of the plant species in database. The genomes of the given species are included in the default setting of ConCysFind. The phylogenetic tree of the selected 21 species was constructed following the Tree of Life Web Project [19] (https://tolweb.org/tree/). The species are depicted as leaves with taxon description at the inner nodes. Here, the edges do not relate to evolutionary distances. Figure 2. Flow diagram of ConCysFind. Depicted are the working steps of the pipeline tool ConCysFind, sub-sectioned by the category of process. Input can be a single protein sequence in single letter code or multiple protein queries as UniProt ID and description in.tsv format. Optionally to the default database, a custom database of 21 species can be used. Each protein sequence 1…i is BLASTed for each species against the database resulting in a maximum of x BLAST hits per sequence i. A nine-times iteration is executed, eliminating close blast hits that do not carry the query AA with a BLAST score cut-off of 0.8 to obtain the nine best BLAST hits per species. A multiple alignment of each sequence i and all species is generated for each stored BLAST sequence. Following the multiple alignments, a phylogenetic tree for the whole protein sequence of each sequence i is constructed and subsequently the p value and score for each analysed AA is calculated. Lastly, for each AA a phylogenetic tree representing the individual conservation-score and p value is drawn. The outputs of the alignments and results are stored as.txt and the phylogenetic trees as.png in a subfolder. Figure 3. Output example of phylogenetic trees with conserved cysteines of known redox targets. (A) Phylogenetic tree of fully conserved Cys167 of the SAL1 phosphatase, a previously characterised target of functional oxidation and disulphide formation [24]. (B) Partial conservation of Cys241 of chloroplast redox sensor 2-cysteine peroxiredoxin in 21 plant species. Figure 4. Phyl [file 12859_2020_3749_MOESM1_ESM.pdf]

**Additional Methods and Handbook for**

# ConCysFind: A pipeline tool to predict conserved amino acids of protein sequences across the plant kingdom.

**Moore, M.[1§], Wesemann, C.[1§], Gossmann, N.[1], Sahm, A.[1], Krüger, J.[2], Sczyrba, A.[2] and Dietz KJ.[1*]**

[1] Biochemistry and Physiology of Plants, Bielefeld University, 33501 Bielefeld, Germany.

[2] Computational Biology Group, Leibniz Institute on Aging – Fritz Lipmann Institute, Jena, Germany

[3] Computational Metagenomics, Bielefeld University, 33501 Bielefeld, Germany.

§ both authors have contributed equally

## Methods

### Cloning and heterologous expression

The complete coding sequence of eRF1-1 (At5g47880.1) was amplified via PCR with specific add-on primers (fw: ATATAGCTAGCATGGGAGACAAAAACGATGACGAC, rv: ATATAGCGGCCGCTTCCGAATCATCATCAAGAGC) and cloned into the pET23a(+) vector (Merck, Darmstadt, Germany) after enzyme restriction with NheI and NotI (NEB, Frankfurt, Germany). Cys-to-Ser variants were obtained by mutagenesis PCR of the complete construct containing the wildtype eRF1-1-CDS with primers inserting a single base substitution (C126S, fw: CCTTTCTGACAACAAGTTTC, rv: TTGTTGTCAGAAAGGTAGAG; C388S, fw: TGGTTCTGCGTTAGAGTTTG, rv: CTCTAACGCAGAACCAAAAC; C404S, fw: TCAGTTTTCCAGAGGGTTTG, rv: CCAAACCCTCTGGAAAACTG). Constructs were ligated and treated with DpnI (NEB, Frankfurt, Germany), prior to transformation in *E. coli* strain Dh5α for clone selection.

Heterologous expression in *E. coli* strain Nico21 (DE3) was induced with 100 µM Isopropyl-β-D-thiogalactopyranosid and conducted for 4 h at 37°C and constant shaking.

**Affinity purification**

Cells were disrupted in lysis buffer (50 mM $NaH_2PO_4$, 300 mM NaCl, 10 mM imidazole- pH 8.0). $His_6$-tag based affinity purification was performed with 2.5 ml Roti®garose-His/Ni beads column (Roth, Karlsruhe, Germany) by subsequent application of 50 ml washing buffer I (50 mM $NaH_2PO_4$, 300 mM NaCl, 20 mM imidazole- pH 8.0) and 50 ml washing buffer II (50 mM $NaH_2PO_4$, 300 mM NaCl, 30 mM imidazole- pH 8.0), followed by 10 ml elution buffer (50 mM $NaH_2PO_4$, 300 mM NaCl, 250 mM imidazole- pH 8.0) to elute column-bound protein. Subsequent dialysis over night at 4 °C in 50 mM sodium phosphate buffer, pH 7.2 and protein amounts were determined spectrophotometrically via absorbance at 280 nm.

**Redox Titration**

9.0 µg of protein were treated with distinct ratios of $DTT_{red}$ and $DTT_{ox}$ to adjust a redox milieu gradient spanning from fully oxidising (≥250 mV) to fully reducing conditions (≤500 mV). Redox conditions were calculated by employing the Nernst equation as described in Ströher *et al.* [1]. All samples were incubated for 30 min at room temperature, before alkylation treatment with 167 mM iodacetamide for 30 min at room temperature in the dark.

**SDS-PAGE, Western Blot and immunodetection**

Discontinuous, non-reducing SDS-PAGE was performed with a 6 % (w/v) acrylamide stacking gel and 8 % (w/v) acrylamide separating gel. The protein was transferred onto a nitrocellulose membrane (Amersham™ Protran™ 0.45 µm, GE Healthcare, Germany) via electroblotting. The membrane was blocked with 1.5 % (w/v) milk powder in 1xTBS for 1 h at room temperature and subsequently incubated with anti-$His_6$ antibody (1:5000 in blocking solution, Invitrogen, Carlsbad, USA) over night at 4°C. After washing steps in 1xTBS and application of anti-mouse peroxidase-coupled secondary antibody (1:50000 in blocking

solution, Sigma-Aldrich, Munich, Germany), $H_2O_2$- and peroxidase reaction-dependent luminescence was started using the Amersham™ ECL Select™ Western Blotting detection reagent kit (GE Healthcare, Germany) and captured on x-ray film.

**Handbook for ConCysFind**

The BibiServ2 platform offers online usage of the pipeline tool in a four-step process.

The first step allows the selection of single or multiple protein analyses. For multiple protein analysis, select the .tsv input function and for single protein analysis the sequence input function.

Step two implements the upload of an input sequence. The upload options for multiple protein analysis are .tsv file, copy and paste or the AWS support. The accepted format for all cases is a UniProt ID in the first column and protein description in the second column, exemplarily displayed when selecting the example button. A description text longer than 100 characters will be trimmed. The same upload options are available for single protein analysis, with amino acid sequence (without UniProt ID) as accepted input format in single letters, also exemplarily shown by selecting the example button. The boundary length of the individual sequences ranges from 20 <= INT <= 10,000.

Step three manages the customization of the pipeline tool parameters. For multiple protein analysis, changeable parameters are: 'Searched amino acid', 'Maximal count of BLAST results', 'BLAST E-Value threshold', 'Substitution BLOSUM matrix', 'Gap open penalty', 'Gap extension penalty', 'Amino acid score threshold' and 'P-Value threshold'. All parameters are explained in section "Parameters" in detail. The same parameters are selectable for single protein sequence analysis, with the addition of the parameters 'Protein sequence description' and 'searched position'. The protein sequence description allows for naming the input sequence, while 'searched position' allows to select an analysis for all

occurrences of the selected amino acid (by setting searched position as “0”). Analysis of a specific amino acid position is possible by selecting a position within the query amino acid sequence (e.g. for Cys at amino acid sequence position 2: “2”). The given position should cover the under “Searched amino acid” specified amino acid. In case of a specified position, the program iterates the working steps of the Blast section until a maximum of 9 iterations is completed (compare Additional Figure 1). This allows the comparison of the most similar homologues between the species with occurring specific amino acids in order to account for random substitutions due to poor sequencing coverage, or multiplication events.

Step four gives a download option for the results from BiBiServ2 as well as an upload option to an AWS Bucket. The pipeline tool is started by selecting “start calculation”.

The results can either be displayed or downloaded as plain text or as compressed results.zip archive, comprising the phylogenetic trees, report.log, RESULT_ALIGNMENT.txt, RESULT_SCORE.txt and RESULT_SCORE.xls.

To display the phylogenetic trees, we recommend using the generated newick strings from the phylogenetic tree folder with the online tool Phy.fi (https://services.birc.au.dk/phyfi/) [2].

**Offline version**

The offline version of ConCysFind is a command-line-based pipeline tool written in Java (Java RE 8 or newer) and runs on Linux, Mac and Windows OS. This chapter describes the use for Windows OS. Download ConCysFind from BiBiServ2 (https://bibiserv.cebitec.uni-bielefeld.de/concysfind?id=concysfind_download) as the fat jar file ConCysFind.jar and the default database in a .zip format. We recommend downloading and unzipping all files into a dedicated new folder for ConCysFind. In addition, blastp (2.7 or newer, as part of blast+) is needed to be installed and an executable file (.exe for windows OS) present in the dedicated ConCysFind folder.

The default database consists of the file uniport_alltaxseq.fasta; custom databases can be generated by assembling the proteome sequences of all species of interest into a .fasta file following the UniProt annotation. The .fasta file is converted into a blastable database by blast+ running the following command line:

makeblastdb -in <FASTA FILE> -dbtype prot

This generates the additional .phr, .pin and .psq files needed for blastp to access the database.

ConCysFind is executed by using the command line:

java -jar ConCysFind.jar -in <INPUT QUERY.TSV FILE> -database <DATABASE NAME> <parameter 1> <parameter 2>…

There is no predetermined order for input parameter options. In the following, all parameters for input and output data supported by the ConCysFind are described.

**Parameters**

Protein sequence description. Description of the single input protein sequence. Setting is optional. A description text longer than 100 characters will be abbreviated.

Searched position. This function allows analysis of the conservation for a particular amino acid position of your single query sequence. The value must reflect an existing position in the query sequence and the amino acid that is searched for. The default setting ”0” calculates the degree of conservation for all given amino acids. Maximum number of amino acid position equals maximum sequence length of 10000, higher values are not allowed.

Searched amino acid. Specification of analysed amino acid for calculating the conservation. Available amino acids are cysteine, tryptophan, serine, threonine, tyrosine and methionine with cysteine set as default.

Maximal count of BLAST results. Maximal count of BLAST results per species, which will be filtered. Boundaries are 500 <= VALUE <= 100000 with the default of 20000.

BLAST E-Value threshold. E-Value threshold for BLAST results. Boundaries are 0.0 < VALUE <= 10.0. Default is 0.0000000001 or rather 1E-10.

Gap open penalty. Gap open penalty for multiple alignment. Boundaries are 1 < VALUE <= 100 with the default of 12.

Gap extension penalty. Gap extension penalty for multiple alignment. Boundaries are 1 < VALUE <= 100 with the default of 1.

Amino acid score threshold. Following the hypothesis that the amino acid in query is probably conserved when the amino acid score is higher than the given threshold. Boundary is 0.1 <= VALUE <= 0.9 with the default of 0.5.

P-value threshold. The amino acid in query is probably conserved when the amino acid's corresponding P-value is lower than the given threshold. Boundaries are 0.1 <= VALUE <= 0.9 with default of 0.5.

Substitution BLOSUM matrix. Substitution cost-matrix for alignment calculation. Different cost matrixes from blosum30 to blosum100 selectable. For best possible alignment resolution, consider the fact that lower matrixes are used for sequences where lower sequence similarity is expected, higher matrix for higher expected similarity. Default matrix is blosum62.

**Additional Output**

The result of ConCysFind is offered as a to .zip file compressed archive for download. The archive contains following files: RESULT_ALIGNMENTS.txt containing the created multiple alignment(s) and RESULT_SCORES.xls containing a table with query information, scores and P-Values.

For every query sequence a phylogenetic tree of all analysed AA is constructed and stored in a Phylogenetic Tree folder. The leaves describe the species of a homologue, with the name marked green for conserved AA and else marked red. The query leaf is marked blue.

**References**


1. Ströher E, Wang X-J, Roloff N, Klein P, Husemann A, Dietz K-J. Redox-dependent regulation of the stress-induced zinc-finger protein SAP12 in Arabidopsis thaliana. Mol Plant. 2009;2:357–67. doi:10.1093/mp/ssn084.
2. Fredslund J. PHY.FI: fast and easy online creation and manipulation of phylogeny color figures. BMC Bioinformatics. 2006;7:315. doi:10.1186/1471-2105-7-315.

# Additional Figures and Tables

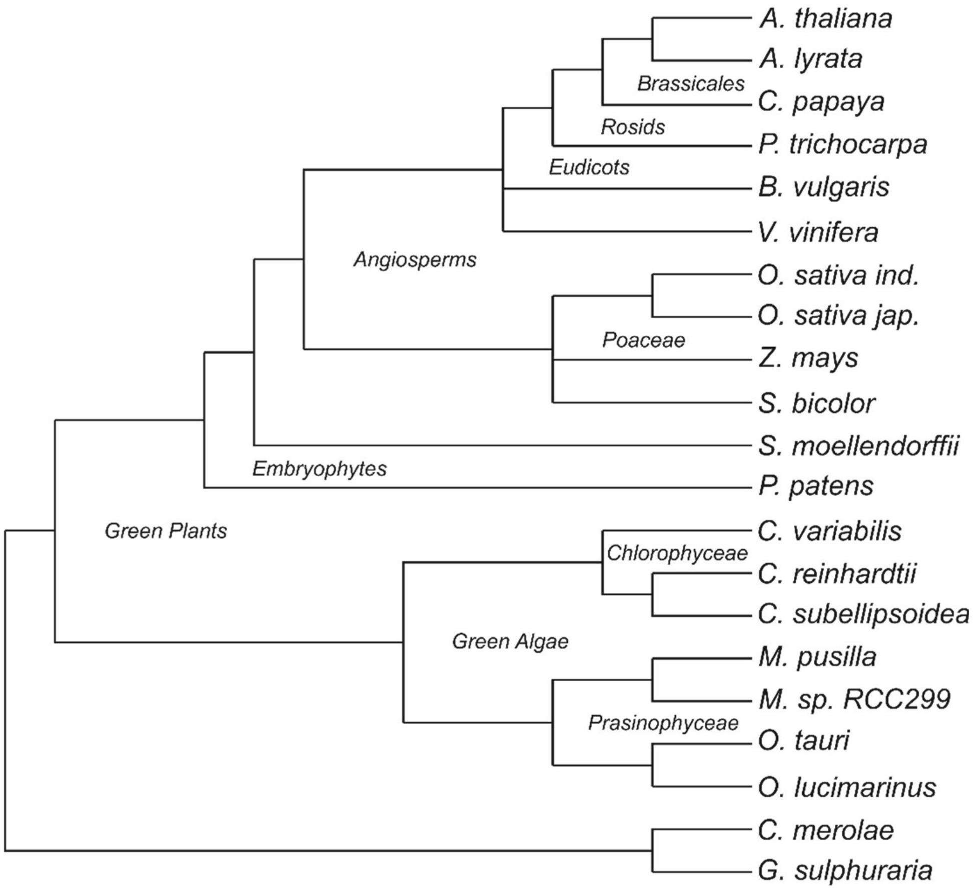


**Additional Figure 1**

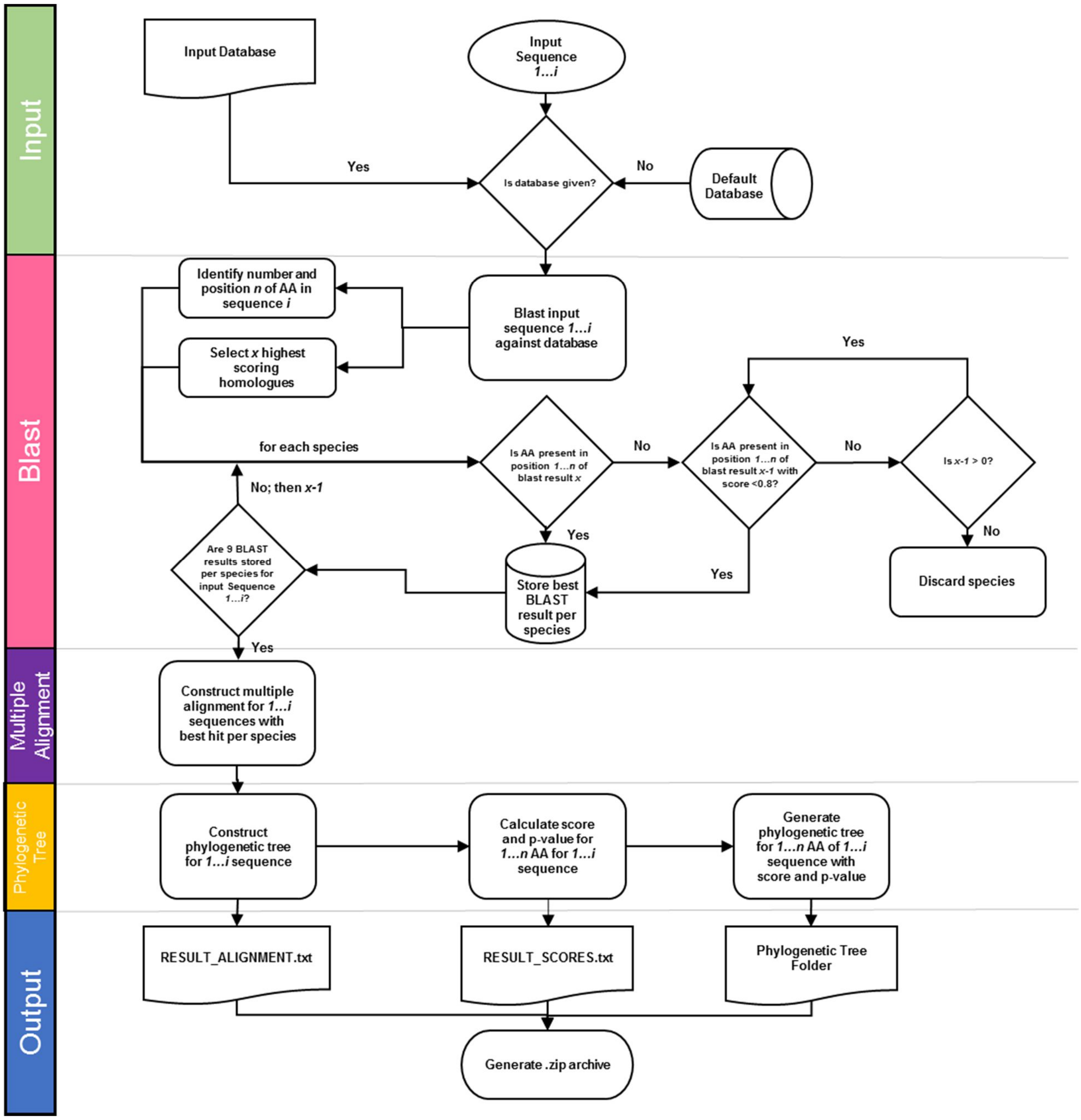


**Additional Figure 2**

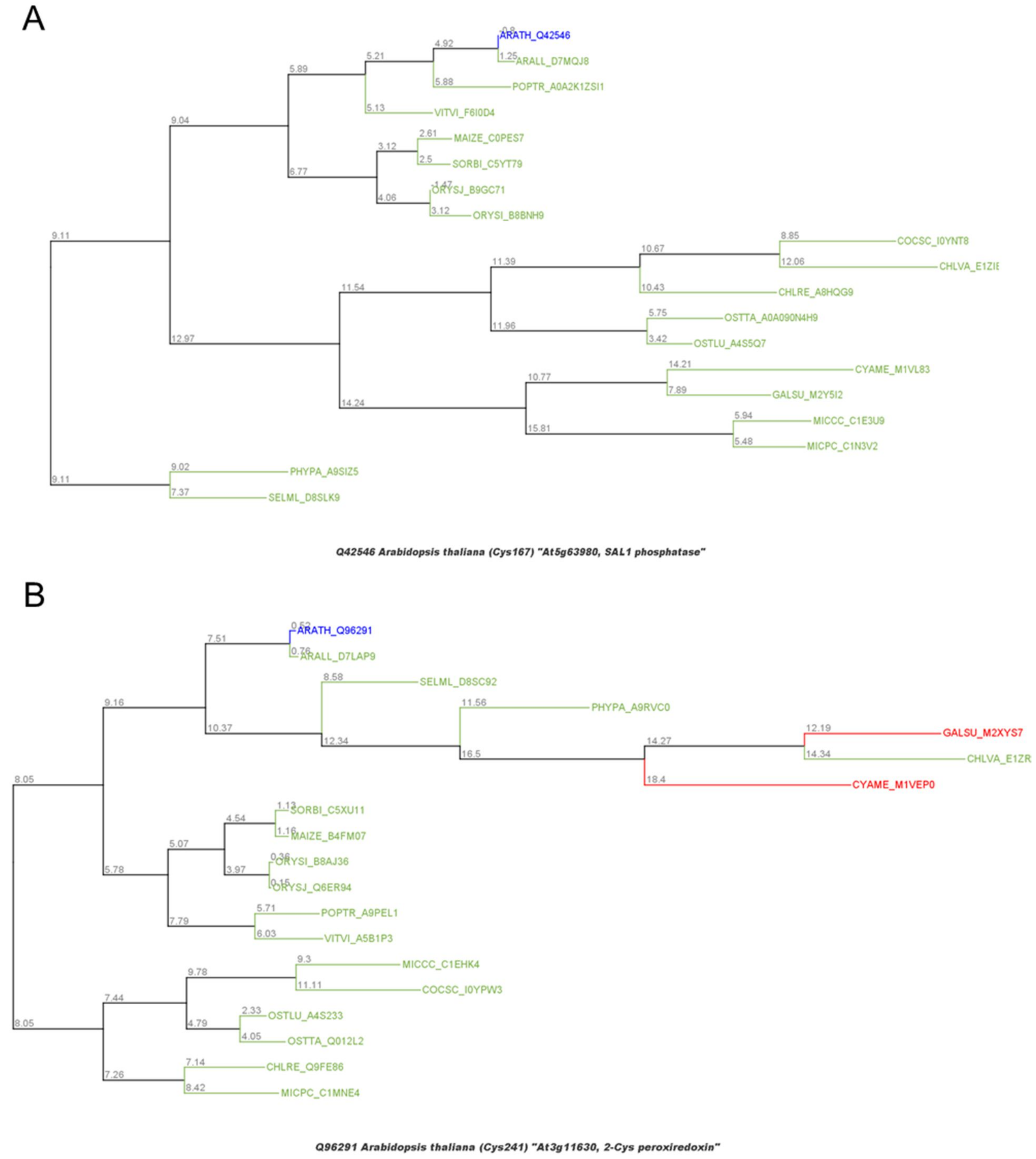


**Additional Figure 3**

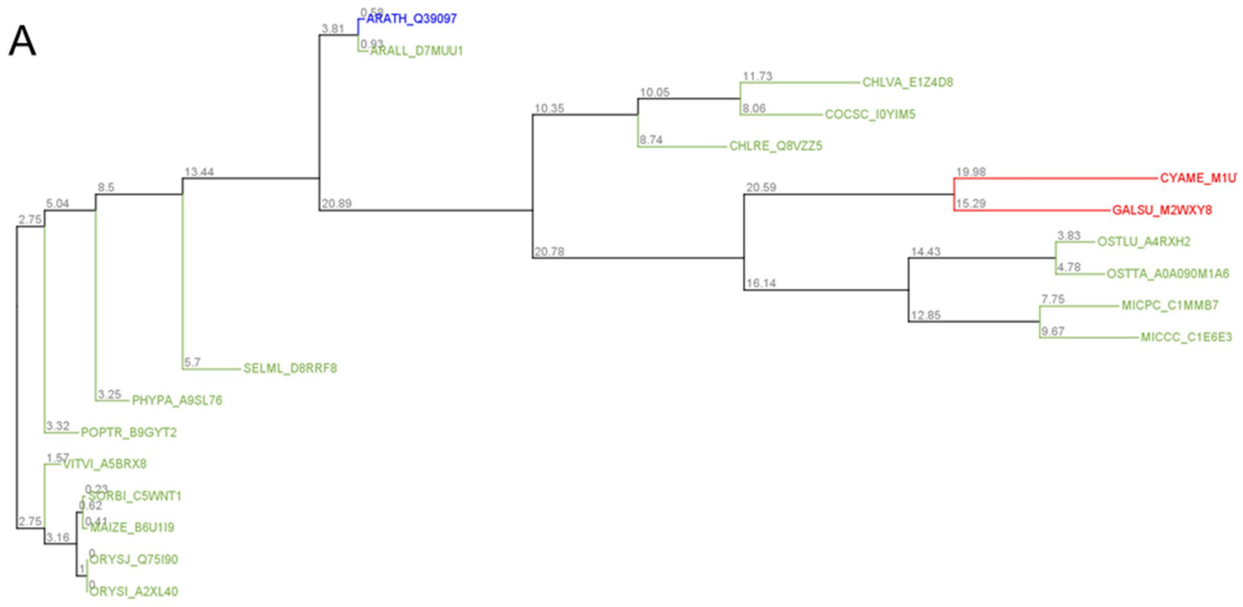


***Q39097 Arabidopsis thaliana (Cys388) "At5g47880, ERF1-1, Encodes a eukaryotic release factor 1"***

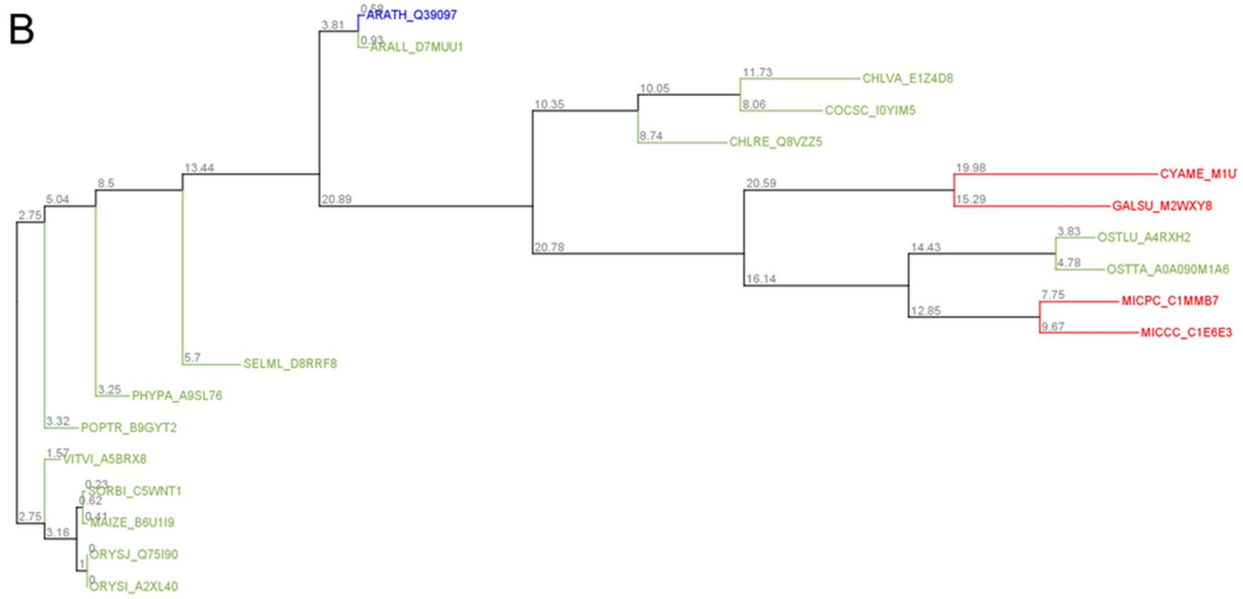


***Q39097 Arabidopsis thaliana (Cys404) "At5g47880, ERF1-1, Encodes a eukaryotic release factor 1"***

# Additional Figure 4

**Additional Table 1**

See separate file Additional Table 1.xlsx

## Additional Table 2

| | |
|---|---|
| O22173 | At2g23350, PABP4 |
| P42731 | At4g34110, PABP2 |
| O64380 | At1g22760, PABP3 |
| Q05196 | At1g71770, PABP5 |
| O04319 | At3g16380, PABP6 |
| Q9FXA2 | At1g49760, PABP8 |
| Q9ZQA8 | At2g36660, PABP7 |
| F4HT49 | At1g34140, PABP1 |
| P0DH99 | At1g07940, GTP binding Elongation factor Tu family protein |
| Q8W4H7 | At1g07930, GTP binding Elongation factor Tu family protein |
| Q0WL56 | At1g07920, GTP binding Elongation factor Tu family protein |
| Q8GTY0 | At5g60390, GTP binding Elongation factor Tu family protein |
| Q84WM9 | At5g12110, Elongation factor 1-beta 1 |
| Q9SCX3 | At5g19510, Translation elongation factor EF1B/ribosomal protein S6 family protein |
| O04487 | At1g09640, Translation elongation factor EF1B, gamma chain |
| Q9FVT2 | At1g57720, Translation elongation factor EF1B, gamma chain |
| P48006 | At1g30230, Elongation factor 1-delta1 |
| Q9SI20 | At2g18110, Translation elongation factor EF1B/ribosomal protein S6 family protein |
| F4IRF5 | At2g18720, Translation elongation factor EF1A/initiation factor IF2gamma family protein |
| F4JQU1 | At4g18330, Translation elongation factor EF1A/initiation factor IF2gamma family protein |
| Q8L835 | At1g18070, Translation elongation factor EF1A/initiation factor IF2gamma family protein |
| A8MRC4 | At1g30230, Elongation factor EF-1BB1 |
| Q9ASR1 | At1g56070/At1g56075, Encodes a translation elongation factor 2-like protein |
| F4IPW5 | At2g31060, Elongation factor family protein |
| Q39097 | At5g47880, ERF1-1, Encodes a eukaryotic release factor 1 |
| Q9LPV8 | At1g12920, ERF1-2, Encodes a eukaryotic release factor one homolog |
| P35614 | At3g26618, ERF1-3, eukaryotic release factor |
| Q9XEX2 | At1g65980, Peroxiredoxin-2B |
| Q96291 | At3g11630, 2-Cys peroxiredoxin |
| Q9XFH8 | At3g02730, Thioredoxin F1 |
| Q39241 | At1g45145, Thioredoxin H5 |
| Q42403 | At5g42980, Thioredoxin H3 |
| Q42546 | At5g63980, SAL1 phosphatase |

# Additional Table 3

| Description | Parameter | Default | Condition |
|---|---|---|---|
| database file | -db | *uniprot_alltaxseq* | readable database in BLAST |
| TSV-file | -in | necessary | TSV-format |
| sequence | -seq | necessary | length of sequence < 5000 amino acids |
| sequence pathway | -sfile | necessary | sequence length amino acids, as .txt file |
| sequence description | -descr | 'no description' | length < 100 |
| conserved amino acid | -aa | Cysteine | amino acid |
| position to be analysed (0, if all cysteine are analysed) | -pos | 0 | can only be used with single input format |
| Maximal BLAST hits | -max | 20,000 | 500 < value < 100000 |
| e-value threshold | -ev | $1*10^{-9}$ | 0 < value < 10 |
| Gap open penalty | -gop | 12 | 1 <= value <= 100 |
| Gap extension penalty | -gexp | 1 | 1 <= value <= 100 |
| BLOSUM | -blosum | Blosum 62 | - BLOSUM |
| Score threshold | -sct | 0.5 | 0.1 <= value <= 0.9 |
| p-value threshold | -pvt | 0.5 | 1 <= value <= 0.9 |
